# Middle East Respiratory Syndrome Coronavirus in Bats, Saudi Arabia

**DOI:** 10.3201/eid1911.131172

**Published:** 2013-11

**Authors:** Ziad A. Memish, Nischay Mishra, Kevin J. Olival, Shamsudeen F. Fagbo, Vishal Kapoor, Jonathan H. Epstein, Rafat AlHakeem, Abdulkareem Durosinloun, Mushabab Al Asmari, Ariful Islam, Amit Kapoor, Thomas Briese, Peter Daszak, Abdullah A. Al Rabeeah, W. Ian Lipkin

**Affiliations:** Ministry of Health, Riyadh, Saudi Arabia (Z.A. Memish, S.F. Fagbo, R. AlHakeem, A. Durosinloun, A.A. Al Rabeeah);; Columbia University, New York, New York, USA (N. Mishra, V. Kapoor, A. Kapoor, T. Briese, W.I. Lipkin);; EcoHealth Alliance, New York (K.J. Olival, J.H. Epstein, P. Daszak);; Ministry of Health, Bisha, Saudi Arabia (M. Al Asmari);; EcoHealth Alliance, Dhaka, Bangladesh (A. Islam)

**Keywords:** coronavirus, bats, Middle East respiratory syndrome, MERS CoV, viruses, Saudi Arabia

## Abstract

The source of human infection with Middle East respiratory syndrome coronavirus remains unknown. Molecular investigation indicated that bats in Saudi Arabia are infected with several alphacoronaviruses and betacoronaviruses. Virus from 1 bat showed 100% nucleotide identity to virus from the human index case-patient. Bats might play a role in human infection.

Since Middle East respiratory syndrome (MERS) was described in September 2012, over 90 cases have been reported worldwide, 70 from Saudi Arabia. The incidence of infection with the causative agent, a betacoronavirus (MERS CoV) (*1*), has not been determined; however, the mortality rate among those who received clinical care is ≈65% (*2*). Although instances of human-to-human transmission have been documented between case-patients and others in close contact (including hospital patients sharing rooms, family members, and medical personnel), the sources of infection for most patients remain unknown. Because of sequence similarities between β-CoVs identified in bats and those of MERS CoV isolated from humans, a bat reservoir has been posited (*3*–*5*). Although neither detection of MERS CoV in bats nor contact of human MERS patients with bats have been reported, a role for bats in human infection cannot be excluded because contact can be indirect (mediated through another animal vector or fomites).

## The Study

**Figure 1:**
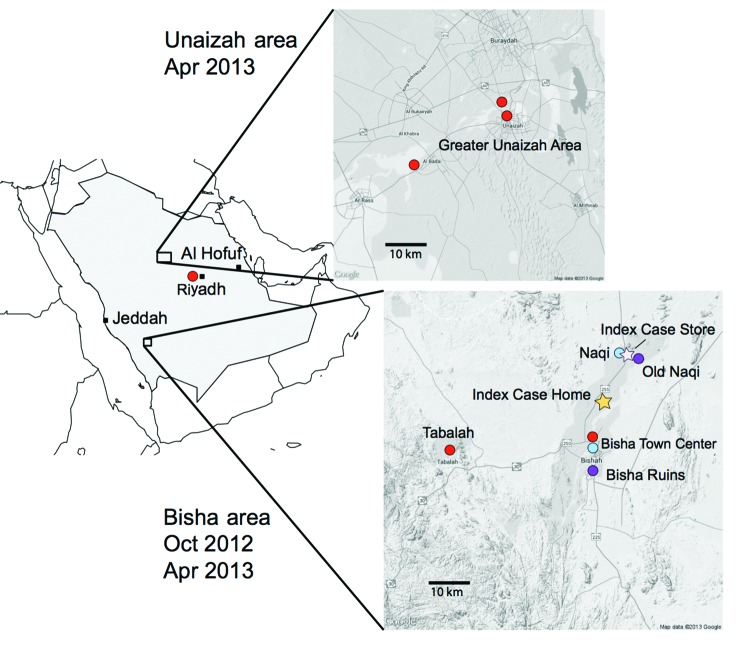
Bat sampling sites and locations of home and workplace of index case-patient with Middle East respiratory syndrome, Bisha, Saudi Arabia.

In October 2012 and April 2013, three agencies collected samples from bats in regions where MERS cases had been identified (Figure 1). The agencies are the Ministry of Health of Saudi Arabia, the Center for Infection and Immunity of Columbia University, and EcoHealth Alliance. 

During the October investigation, the team interviewed the family of an index case-patient in Bisha and collected samples from bats <12 km from his home, in an abandoned date palm orchard, and <1 km from his place of employment, a hardware store that fronted a garden and date palm orchard. Although neither family members nor employees recalled seeing bats, the team observed roosting bats and guano in abandoned wells and ruins within 12 km of his home and insectivorous bats at dusk in the garden behind his store. Over 3 weeks, 96 bats representing 7 species (*Rhinopoma hardwickii*, *Rhinopoma microphyllum*, *Taphozous perforatus*, *Pipistrellus kuhlii*, *Eptesicus bottae*, *Eidolon helvum*, and *Rosettus aegyptiacus*) were captured in mist nets and harp traps, then released after visual speciation and collection of morphometric measurements; wing punch biopsy samples; blood; throat swab samples; and rectal swab samples or fecal pellets. Samples were collected into viral transport medium or lysis buffer.

During the 3-week April investigation, fecal samples were collected on tarps laid out at bat roosting sites in and around Bisha, Unaizah, and Riyadh. Representative animals at each roosting site were captured, identified morphologically, and released after wing punch biopsy samples were collected for speciation by DNA analysis. Samples were collected into cryovials.

All samples were stored in liquid nitrogen and conveyed to Riyadh for storage at −80°C before being transported to Columbia University in New York in dry nitrogen. The October 2012 shipment was inadvertently opened at customs in the United States and sat at room temperature for 48 hours before transfer to Columbia University; at arrival, all samples had thawed. The April 2013 samples arrived intact.

Total nucleic acid was extracted from samples by using the NucliSENS easyMAG system (bioMérieux, Durham, NC, USA) and subjected to 8 PCRs with primers and protocols designed to amplify regions within the helicase, RNA-dependent RNA polymerase (RdRp), and nucleocapsid or envelope proteins of CoVs (*6*–*9*). Products were sequenced and analyzed for similarity to GenBank database entries by using the BLASTn and BLASTx programs (www.ncbi.nlm.nih.gov/blast/Blast.cgi). Primer sequences are shown in Table 1. The identity of bat species yielding specific viral products was determined by amplifying and sequencing a fragment of the cytochrome B gene (*10*). All visual classifications of species were confirmed except for that of *T. perforatus* bats. There is no reference sequence for *T. perforatus* bats in GenBank. However, because the closest reference sequence was from *T. nudiventris* bats, at 84% identity we presume that the product represents bona fide *T. perforatus* bat cytochrome B gene sequence. Representative cytochrome B sequences have been uploaded to GenBank (accession nos. KF498635–KF498641).

**Table 1:** PCRs and primers used in CoV detection*

PCRs (reference)	Primers, 5′→3′	Nested fragment size, region (primer locations on the reference) genome)†	Type of CoV (no.)
Nested pan-CoV-I (*6*)	PLQ-F1, CGTTGGIACWAAYBTVCCWYTICARBTRGG	≈400 nt, RdRp (18310–187450)	α-CoV (8), β-CoV (1)
	PLQ-R1, GGTCATKATAGCRTCAVMASWWGCNACATG		
	PLQ-F2, GGCWCCWCCHGGNGARCAATT		
	PLQ-R2, GGWAWCCCCAYTGYTGWAYRTC		
Nested pan-CoV-II (*7*)	WT-COV-F1, GGTTGGGAYTAYCCHAARTGTGA	≈430 nt, RdRp (15260–15700)	α-CoV (5), β-CoV (2)
	WT-COV-R1, CCATCATCASWYRAATCATCATA		
	WT-COV-F2, GAYTAYCCHAARTGTGAYAGAGC		
	WT-COV-F3, GAYTAYCCHAARTGTGAUMGWGC		
Hemi-nested RdRp-sequence assay (*9*)	EMC-SeqRdRP-Rev, GCATWGCNCWGTCACACTTAGG	≈230 nt, RdRp (15048–15290)	α-CoV (2), β-CoV (1)
	EMC-SeqRdRP-Fwd, TGCTATWAGTGCTAAGAATAGRGC		
	EMC-SeqRdRP-Rnest, CACTTAGGRTARTCCCAWCCCA		
Hemi-nested N-sequence assay (*9*)	EMC-SeqN-Fwd, CCTTCGGTACAGTGGAGCCA	≈280 nt,N seq (29,549–29,860)	–
	EMC-SeqN-Rev, GATGGGGTTGCCAAACACAAAC		
	EMC-SeqN-Fnest, TGACCCAAAGAATCCCAACTAC		
Nested CII-pan-CoV-III	NM-CoV-2F1, ACWGTTCARGGICCWCCIGG	≈355 nt, helicase (17,060–17,410)	β-CoV (2)
	NM-CoV-2F2, GTTCARGGGCCWCCGGGNAC		
	NM-CoV-2R1, GGCAGCTGWGCWGGRTCICCNACRTA		
	NM-CoV-2R2, AGCTGWGCWGGRTCGCCIACRTANAC		
Nested CII-MERS-RdRp	NM-HCOV-F1, GTGCTAAGAATAGAGCTCGCACT NM-HCOV-F2, AGAGCTCGCACTGTTGCAGGC	≈190 nt, RdRp (15068–15249)	β-CoV (1, MERS CoV)
	NM-HCOV-F2, AGAGCTCGCACTGTTGCAGGC		
	NM-HCOV-R1, ACCCATAAGATGCGGATTATCAAC		
	NM-HCOV-R2, TGCGGATTATCAACATCTTTGTAC		
Hemi-nested CII-MERS N sequence	NM-NSeq-F-1, ACTTCCTTCGGTACAGTGGAGC	≈170 nt, N seq (29545–29713)	–
	NM-NSeq-R-1, GGCACTGTTCACTTGCAATC		
	NM-NSeq-R-2, GGAGGTTCAGACATTTGGTCT		
upE and ORF1b real-time assays (*8*)	upE-Fwd: GCAACGCGCGATTCAGTT	Upstream of E gene and ORF 1b	–
	upE-Prb: FAM-CTCTTCACATAATCGCCCCGAGCTCG-TAMRA		
	upE-Rev: GCCTCTACACGGGACCCATA		
	ORF1b-Fwd: TTCGATGTTGAGGGTGCTCAT		
	ORF1b-Prb: FAM-CCCGTAATGCATGTGGCACCAATGT-TAMRA		
	ORF1b-Rev: TCACACCAGTTGAAAATCCTAATTG		

* CoV, coronavirus; MERS, Middle East respiratory syndrome; RdRp, RNA-dependent RNA polymerase; –, not applicable; ORF, open reading frame. †Primer locations are based on human β-CoV 2c EMC/2012, complete genome (GenBank accession no. JX869059).

Table 1 indicates the CoV genera identified by using individual primer sets. As anticipated, pan-CoV assays detected α- and β-CoVs. One assay specific for MERS CoV (*9*) also detected α-CoVs. This finding reinforces the need for sequence confirmation of PCR products. Table 2 indicates the CoV species identified with respect to location, sample type, and bat species. CoV sequences were amplified from rectal swab samples or fecal pellets and from roost feces but not from serum, throat swab samples, or urine. Alpha CoV sequences were amplified more frequently than β-CoV sequences (223 vs 4). Whereas α- and β-CoV sequences were amplified from CoVs from *T. perforatus*, *E. helvum,* and *R. hardwickii* bats, only alpha sequences were amplified from CoVs from *P. kuhlii* bat samples.

**Table 2:** CoVs detected in bats, Saudi Arabia*

Bat family, genus, species	Location	No. Bats	No. samples tested (no. positive)					Total samples (n = 1,003)	Total positive samples (n = 227) (closely related CoVs)†
			Throat swab	Fecal pellets	Urine	Serum	Roost feces		
October 2012									
Emballonuridae									
* Taphozous perforatus*	Bisha ruins	29	29 (0)	25 (2)	8 (0)	22	10 (1)	94	1 β-Cov (1 MERS novel CoV) and 2 α-CoVs (1 bovine respiratory CoV, 1 Kenya bat CoV BtKY86)
Pteropodidae									
* Eidolon helvum*	Bisha town center	25	25 (0)	25 (5)	13 (0)	19	–	82	1 β-CoV (1 *Eidolon* bat CoV-HKU1) and 4 α-CoVs (4 Kenya bat CoV BtKY86)
* Rousettus aegyptiacus*	Bisha town center	3	3 (0)	3 (0)	1 (0)	2	–	9	–
Rhinopomatidae									
* Rhinopoma hardwickii*	Naqi and Old Naqi	36	36 (0)	35 (0)	4 (0)	–	15 (0)	90	–
* Rhinopoma microphyllum*	Old Naqi	1	1 (0)	1 (0)	–	–	–	2	–
Vespertilionidae									
* Eptesicus bottae*	Bisha ruins	1	1 (0)	1 (0)	1 (0)	–	32 (0)	35	*–*
* Pipistrellus kuhlii*	Bisha ruins	1	1 (0)	1 (0)	–	–	–	2	–
April 2013									
Rhinopomatidae									
* Rhinopoma hardwickii*	Greater Bisha area	–	–	–	–	–	209 (93)	209	2 β-CoVs (2 canine respiratory CoVs) and 91α-CoVs (5 canine CoVs, 2 *Miniopterus* bat CoVs, 84 *Chaerephon* bat CoV)
* Taphozous perforatus*	Bisha ruins	–	–	–	–	–	203 (0)	203	–
Vespertilionidae									
*Pipistrellus kuhlii*	Greater Unaizah area	9	9 (0)	–	–	–-	263 (126)	277	126 α-CoVs (69 alphaCoV P.kuh-Spain, 3 canine CoVs, 37 bat CoV P.pyg/Germany, 1 human CoV NL63, 2 *Rousettus* bat CoV HKU10, 11 Porcine epidemic diarrhea virus, 2 *Cardioderma* bat CoVs, 1 *Hipposideros* bat CoV HKU10)
	Greater Riyadh area	5	5 (0)	–	–	–	–	–	–

*CoV, coronavirus; MERS, Middle East respiratory syndrome; –, not applicable.  †Based on BLASTn (www.ncbi.nlm.nih.gov/blast/Blast.cgi).

**Figure 2:**
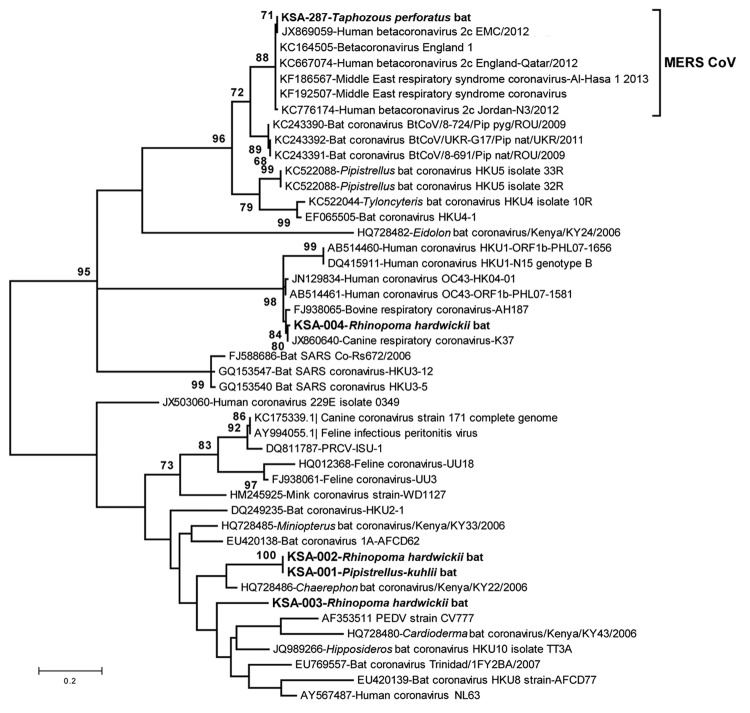
Phylogenetic tree showing genetic relatedness between coronaviruses identified in bat samples from Saudi Arabia (**boldface**), MERS coronaviruses, and other published coronavirus sequences available in GenBank. The maximum-likelihood tree of partial RNA-dependent RNA polymerase gene (nt position 15068–15249 of GenBank accession no. JX869059) was constructed by using the Tamura-Nei model with discrete gamma rate differences among sites as implemented in MEGA 5.2 (www.megasoftware.net). Each branch shows the GenBank accession number followed by a brief description of the sequence used. Scale bar indicates nucleotide substitutions per site. MERS, Middle East respiratory syndrome; CoV, coronavirus; SARS, severe acute respiratory syndrome; KSA, Kingdom of Saudi Arabia.

CoV sequences were amplified from 220 of 732 roost feces samples and 7 of 91 rectal swab samples or fecal pellets. A product obtained by PCR amplification of nucleic acid from a fecal pellet of a *T. perforatus* bat captured in October 2012 in Bisha showed 100% nt identity to the human β-CoV 2c EMC/2012 cloned from the index case-patient in Bisha. A phylogenetic analysis of CoVs obtained in this study is shown in Figure 2. CoV sequences have been uploaded in GenBank (accession nos. KF493884–KF493888).

## Conclusions

A wide range of CoV species are circulating among bats in Saudi Arabia. Although the prevalence of CoVs was high (≈28% of fecal samples), MERS CoV was found in only 1 bat. A 3.5% MERS CoV infection rate (n = 29; 95% CI 0–20%) in *T. perforatus* bats is low compared with that for severe acute respiratory syndrome–like CoV in rhinolophid bats in China (10%–12.5%) but consistent with CoV prevalence among bats in Mexico (*4*). Furthermore, the sensitivity for viral nucleic acid detection in samples collected in October 2012 was probably reduced because of failure in cold chain transport. Whereas 219 (32%) of 675 of fecal pellets collected in April revealed a CoV sequence by PCR, only 8 (5%) of 148 of rectal swab samples or fecal pellets collected in October were positive by the same assays. We were unable to recover additional sequences beyond the 190-nt RdRp fragment represented in Figure 2 but are confident in the fidelity of the finding. First, although RdRp is a conserved portion of the CoV genome, there is no precedent for 100% identity of a bat sequence with a human MERS CoV sequence. Second, when this work began we did not have cultured MERS CoV, human MERS samples, or MERS CoV cDNA in the laboratory at Columbia University where samples were removed directly from the tubes in which they were collected in the field for nucleic acid extraction, PCR, and sequence analysis. Third, the only MERS-positive signal was obtained in PCR analysis of the *T. perforatus* bat captured in Bisha near the home and workplace of the MERS index case-patient used to generate the human β-CoV 2c EMC/2012 sequence.

Bats are reservoirs of several viruses that can cause human disease, including rabies, Hendra, Nipah, Marburg, severe acute respiratory syndrome CoV, and probably Ebola viruses (*11*–*14*). Cross-species transmission from bats to humans can be direct, through contact with infected bats or their excreta, or facilitated by intermediate hosts (*15*). Bat CoVs are typically host specific; however, MERS-related CoVs have reportedly been found in many bat families, including Vespertillionidae, Molosidae, Nyteridae, and now Emballonuridae (sheath-tailed bats) in Africa, the Americas, Asia, and Europe. We sampled only a small sample of bats in Saudi Arabia. Nonetheless, given the rarity of MERS CoV sequences detected by our survey and the broad distribution of MERS cases throughout the Middle East, we speculate that there are probably other hosts. Future work should investigate additional bat and other wildlife species and domestic animals for CoV infection and potential linkage to human disease.
